# The effect of TNF treatment uptake on incident hospital admission in Western Australia

**DOI:** 10.1186/s12969-023-00810-1

**Published:** 2023-03-28

**Authors:** Erin Kelty, Ebony Quintrell, David B. Preen, Prue Manners, Johannes Nossent

**Affiliations:** 1 School of Population and Global Health, The University of Western Australia, 35 Stirling Hwy (M503), PerthCrawley, WA 6009 Australia; 2 Telethon Kids Institute, Perth, Australia; 3 Rheumatology Section, Division Medicine, Medical School, The University of Western Australia, Perth, Australia; 4 Department of Rheumatology, Sir Charles Gairdner Hospital, Perth, Australia

**Keywords:** Juvenile idiopathic arthritis, Hospitalisation, Prevalence, TNF inhibitors, Arthrocentesis

## Abstract

**Objective:**

Treatment strategies for juvenile idiopathic arthritis (JIA) have shifted significantly over the last 20 years. We examined the effect of the introduction of government-subsidised TNF inhibitor (TNFi) treatment on incident hospitalisation for JIA.

**Methods:**

Western Australian (WA) hospital data were used to identify patients < 16 years hospitalised with JIA between 1990 and 2012. Changes in the number of patients with an incident hospitalisation, overall admissions and admissions for joint aspiration were examined using join-point regression TNFi dispensing data from 2002–2012 was used to describe defined daily doses (DDD)/1000 population/day.

**Results:**

We included 786 patients (59.2% girls, median age 8 years) with a first-time admission with JIA. The annual incident admission rate was 7.9 per 100,000 person-years (95%CI: 7.3, 8.4) which did not change significantly between 1990 and 2012 (annual percentage change (APC): 1.3, 95%CI: -0.3, 2.8). Annual hospital-based prevalence of JIA reached 0.72/1000 in 2012. DDD for TNFi usage rose steadily from 2003 indicating TNFi usage by 1/2700 children in 2012, while overall admission rates (APC 3.7; 95%CI: 2.3, 5.1) and admission rates for joint injections (APC 4.9%; 95%CI: 3.8, 6.0) also increased significantly in that period.

**Conclusion:**

Incident inpatient admission rates for JIA were stable over a 22-year period. The uptake of TNFi was not associated with lower admission rates for JIA, due mainly to an increase in admissions for joint injection. These results indicate a notable but unexpected change in hospital-based management of JIA since the introduction of TNFi therapy in WA, where hospital-based prevalence of JIA is slightly higher than in North America.

**Supplementary Information:**

The online version contains supplementary material available at 10.1186/s12969-023-00810-1.

## Introduction

Juvenile idiopathic arthritis (JIA) designates idiopathic arthritis with onset before the age of 16 years that lasts for at least six weeks [1–3]. JIA often persists into adulthood and can cause substantial long-term physical morbidity [4]. JIA management includes prompt initiation of appropriate drug therapy to improve symptoms and prevent permanent joint damage in addition to allied health support [5, 6]. Increasing evidence supports early aggressive treatment with synthetic and biological disease-modifying anti-rheumatic drugs (DMARDs) to modify the disease course and improve long-term prognosis [7]. Government-subsidised bDMARD therapy has been available in Australia in the form of Etanercept (since 2003), Adalimumab (since 2010) and Tocilizumab (since 2012) for patients with severe active juvenile idiopathic arthritis. In Western Australia (WA), JIA care is generally coordinated by a small number of paediatric rheumatologists in hospital outpatient settings or private rooms. We evaluated whether the availability of TNF inhibitor therapy for JIA since 2003 was associated with a change in hospital admission rates and admitting diagnoses over time.

## Methods

This was a whole-population-level observational study including all patients aged ≤ 15 years with an incident (ie, first-time) hospital admission with JIA between 1 January 1980 and 31 December 2012 in WA. The study made use of the Western Australia Rheumatic Disease Epidemiological Register (WARDER) described elsewhere [8]. In brief, the WARDER is a rheumatic disease research repository that contains routinely collected health data across public and private hospitals in WA over a 30 year period for patients with inflammatory rheumatic diseases. Data from four separate datasets are then linked through a validated process of probabilistic matching and clerical review to provide individual longitudinal health data [9, 10]. For this study, WARDER data were extracted from the WA Hospital Morbidity Data Collection (HMDC) (1980–2015) to identify patients ≤ 15 years who had an incident hospital admission between 1990 and 2012 with a primary or co-diagnosis of JIA, coded using International Classification of Diseases version 9, Clinical Modification (ICD-9-CM) and version 10, Australian Modification (ICD-10-AM). The ICD-9-CM codes used to identify JIA were 714.3, 696.0, 720.0, 720.2, 720.89, and 720.9, while the ICD-10-AM codes were M08.0-M08.9 and M09.0 (with L40.5). Guidelines from the CMS General Equivalence Mappings were applied to translate ICD9 to relevant ICD-10 codes (https://www.cms.gov/medicare/icd-10/2022-icd-10-cm). Basic demographic information including age, sex, Aboriginality, and rurality at the index hospitalisation were obtained from hospital data and admissions with arthrocentesis (ICD9: 81.9, 83.9/ ICD10: 50,124) and infections (ICD9: 000–140/ ICD10: A00-B99) were additionally identified (Supplementary table 1).

### TNFi dispensing data

In Australia, Etanercept was approved for JIA in 2003 and Adalimumab in 2010 with government subsidised TNFi therapy available to JIA patients following an application by a recognised specialist to Medicare Australia for access to TNFi treatment. Prescription requires documentation of severe active JIA not responding to, or the patient being unable to tolerate, Methotrexate treatment alone or in combination with another DMARD and oral or intra-articular corticosteroids (see https://www.servicesaustralia.gov.au/organisations/health-professionals/forms/pb060 for details). Annual WA-specific data from the Pharmaceutical Benefits Scheme (PBS) for dispensing (service) and costs (benefits) for the two TNFi approved for JIA treatment (Etanercept, Adalimumab) were obtained from the Australian Government Department of Human Services (http://medicarestatistics.humanservices.gov.au/statistics/pbs_item.jsp) using the disease-specific authority codes for TNFi treatment for JIA in the period 2004–2015 (Supp Table 2).

TNFi dispensing was classified by their Anatomical Therapeutic Chemical (ATC) code and for each study year the corresponding WHO-approved DDD was calculated. DDD was then expressed as the number of DDDs/1,000 children/day to estimate the proportion of JIA patients within the community who received TNFi.

### Statistical Analysis

To remove any potential prevalent pool effect we selected patients with a first JIA hospitalisation between 1990 and 2012 and excluded all patients with earlier JIA hospital admissions during a 10-year lookback period. All hospitalisation episodes in patients with a diagnosis of JIA when aged < 16 years were included in estimates of the total number of JIA hospitalisation between 1990 and 2012. To calculate DDD, admission and hospital-based prevalence rates, we obtained population level data by age and gender from the Australian Bureau of Statistics for WA. (https://www.abs.gov.au/statistics/people/population/national-state-and-territory-population/latest-release#data-downloads-data-cubes). Temporal trends from 1990 – 2012 were examined using join-point regression with results expressed as annual percentage change (APC) with 95% confidence intervals (95%CI).

### Ethics

Approvals for this project were obtained from the WA Department of Health Human Research Ethics Committee (HREC) (# 2016/24) and University of Western Australia HREC (approval number RA/4/20/4070).

## Results

### First hospital admission with JIA

**Fig. 1:**
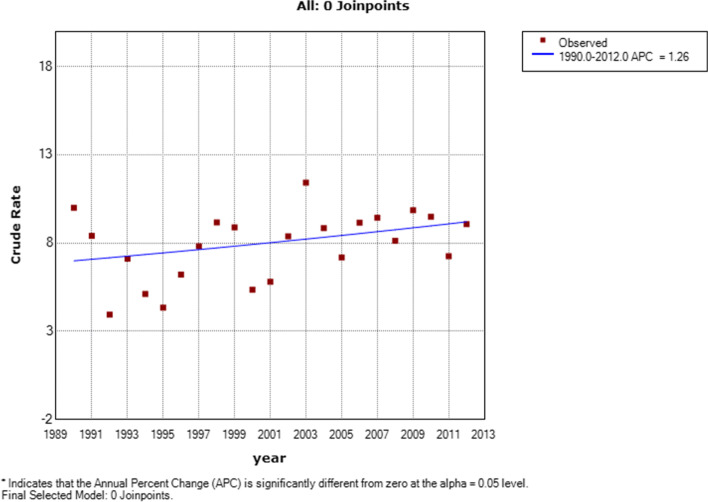
First time hospital admission rates per 100,000 children for juvenile idiopathic arthritis in Western Australia between 1990 and 2012

**Fig. 2:**
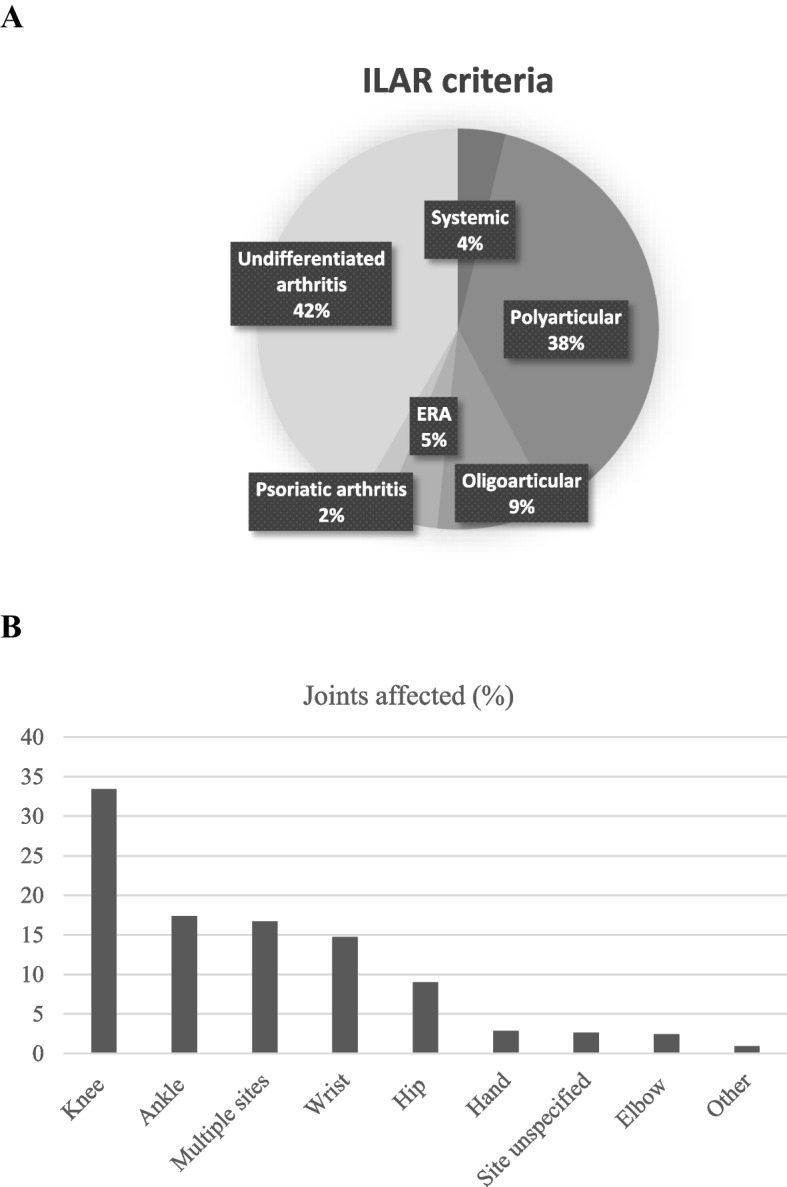
**a** Subtype distribution for patients admitted with juvenile arthritis **b** Distribution of joints affected in patients admitted with ICD-10AM code for juvenile arthritis since 2000 (*n* = 455)

**Fig. 3:**
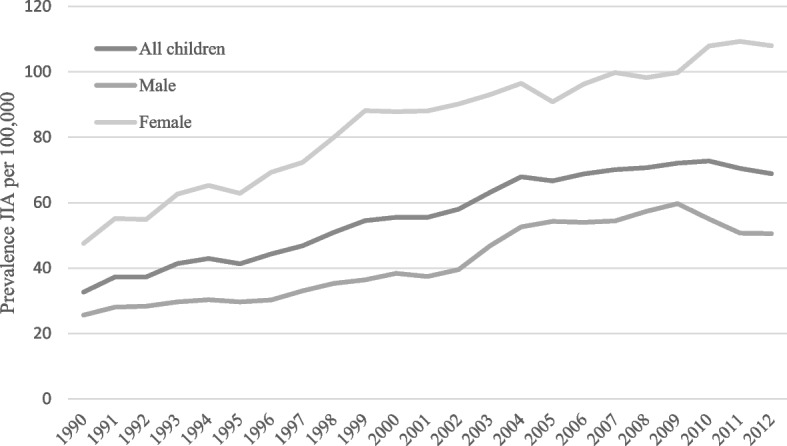
Hospital based prevalence of juvenile idiopathic arthritis in Western Australia in period 1990–2012

Between 1990 and 2012, 786 JIA patients had a first hospital admission at a median age of 8 years (IQR 4–11) and with predominance of female patients (*n* = 465, 59.2%). At the index admission, 82.2% of patients lived in metropolitan regions and 6.1% identified as Aboriginal. Over the study period, 7.9 (95%CI: 7.3, 8.4) per 100,000 children under 16 years were hospitalised for the first time with JIA; 6.3 (95%CI: 5.6, 7.0) per 100,000 in boys and 11.2 (95%CI: 10.2, 12.3) per 100,000 in girls. The JIA first admission rate was stable over the study period (APC: 1.3, 95%CI: 0.3, 2.8, *p* = 0.100) (Fig. 1). JIA was the primary admitting diagnosis in 89.8% (*n* = 706) of incident hospitalisations with the remainder of primary admission diagnoses due to a range of conditions including 18.7% infections and 4% uveitis (Suppl Fig. 1). ICD coding for JIA is not fully compatible with the ILAR classification criteria but, using the CMS General Equivalence Mappings, the majority of admitted JIA patients had polyarticular (38.4%) or undifferentiated JIA (41.6%) (Fig. 2a). Using the 455 admissions with ICD-10-AM coding (as ICD-9-CM does not provide site specific data), lower extremity joints were more frequently affected than upper limb joints (Fig. 2b). Based on the above we estimated a hospital-based point-prevalence for JIA in WA of 72.3 per 100,000 (Fig. 3) in 2012 with prevalence in girls (114.5 per 100,000) more than twice that in boys (52.9 per 100,000).

### All Hospitalisations for JIA

There were a total of 2394 hospital admissions in this JIA cohort over the study period and the hospital admissions increased significantly over time (APC 3.7; 95%CI: 2.3, 5.1. *p* < 0.01). The number of joint aspirations performed in JIA patients increased from 15.2 per 100,000 per year in 1990 to 34.7 per 100,000 per year in 2012. The APC was 4.9% (95%CI: 3.8, 6.0) (Suppl Fig. 2a) with a particular increase in admissions and joint aspiration seen around 2003 (Suppl Fig. 2b).

### TNFi usage

**Fig. 4:**
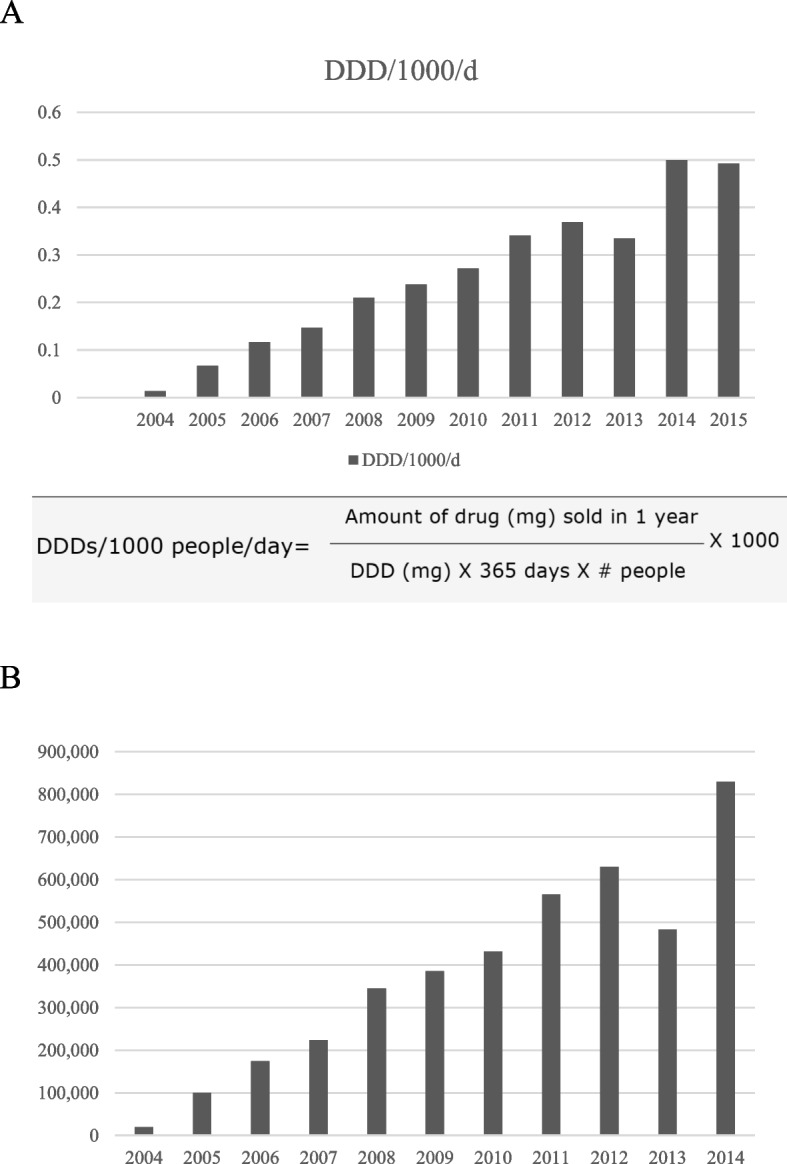
**a**: Define daily dose of TNF inhibitor (Etanercept and Adalimumab) usage for juvenile idiopathic arthritis in Western Australia between 2004 and 2015 **b**: Annual cost (in AUD) of TNF inhibitor (Etanercept and Adalimumab) usage for juvenile idiopathic arthritis in Western Australia between 2004 and 2015

TNFi usage (Fig. 4a) and costs (Fig. 4b) for JIA in WA increased from 2003 onwards. The DDD/1000 /day reached 0.37/1000 in 2012, indicating TNFi usage for JIA by 1 in 2700 children in WA.

## Discussion

This study found no change in the rate of first-time hospital admissions for JIA over 22 years in WA but a significant increase in overall rates for JIA admission and joint injections since 2003, following significant uptake of biological therapy with 1 in 2700 children in WA on TNFi therapy for JIA at the study end date. We also report a hospital-based prevalence of JIA in WA of 72.3/100.000 with levels twice as high for girls as for boys.

The demographics of this Australian group of JIA patients were similar to studies of JIA patients in other countries, where female patients outnumber male patients and average age at diagnosis was 7 years [11–13]. The proportion of children with JIA identifying as Aboriginal was similar to that seen in the general WA community (6.1% versus 5.9%, respectively) [14], suggesting Aboriginal children were not at an increased risk of hospitalisation with JIA. The stable rates for a first-time admission for JIA indicates that the availability of bDMARD for JIA in this century has not impacted on the perceived need for hospital-based management of JIA in WA. As therapeutic guidelines for JIA were unavailable prior to expert based recommendations in 2011 [15], JIA management in the study period was dependent on the training/usual practice of the consulted specialists with usual practice to escalate to DMARD therapy if no improvements were seen after 2–3 months of NSAID monotherapy or earlier with severe disease activity. The stable hospitalisation rate could reflect a local preference for intraarticular joint injections and/or a slow rate of uptake of DMARD initiation and limitations in early access to biologicals as first line therapy [3]. As rates for subsequent admissions were paralleled by an increase in the number of joint aspirations/injections during the disease course since 2003, this points towards an increased reliance on intra-articular corticosteroid injections as stand-alone or adjunct to initiating DMARD therapy. Alternatively, it may relate to the fact that access subsidised TNFi therapy requires active disease in the context of DMARD inefficacy/intolerance and failed corticosteroid administration and /or a lack of resources for outpatient management [3, 16, 17]. While we are unable to tease out the details behind this, the presented data indicate that the costs associated with the increasing TNFi usage in WA have not been offset by reduced hospitalisation rates.

In agreement with other studies, lower limb joints were most frequently affected in this cohort [18, 19]. Based on an admittedly crude approximation of ILAR classification through ICD codes, most patients in this study had polyarticular or unspecified JIA with only 9% recorded to have oligoarticular disease. Oligoarticular presentation is considered the most common subtype of JIA and, although this is not the case in all countries [20–22], patients with oligoarticular JIA in this study may have been (mis)classified as unspecified or are truly underrepresented in this study. As treatment recommendations for oligoarticular but not polyarticular JIA suggest intraarticular corticosteroid injections as first line [3, 15], the number of oligoarticular JIA patients in this study was likely higher than the 9% coded as oligoarticular JIA. Finally, the distribution of affected joints has been the basis for the classification of JIA subtypes [2, 3]. However, as this distribution changes frequently over time [23] and recent studies demonstrate that a large proportion of JIA patients meet classification criteria for idiopathic arthritis in adults, the usefulness for separate phenotypes for childhood arthritis is being questioned [8–10].

The global prevalence of JIA shows considerable variation and while our hospital based data likely underestimate the true population prevalence [24, 25], the 2012 point-prevalence is at the higher end of prevalence estimates reported from other hospital-based studies [12, 26]. However, it remains well below the estimated prevalence reported from a previous cross-sectional Australian study of 12 year-old metropolitan students where approximately 4/1000 were found to have JIA [25], while similar studies from Belgium (in children aged 12–18 years) and a recent study from Brazil (children aged 1–16 years) found community-based prevalence estimates of 1.7 and 2 per 1000 children, respectively [27, 28]. Together these data illustrate that there is a number of patients with JIA that have milder and/or spontaneously remitting JIA, that will not be captured in hospital-based data [29].

Key strengths of this study included the availability of state-wide population-based data in WA, the large number of patients and the longitudinal analysis allowing examination of trends over time. Limitations of this study include the reliance on hospital data, likely resulting in an underestimation of the community prevalence of JIA. Also, validation of administrative data for identifying JIA in Australia has not been carried out, but studies from North America have reported ≥ 90% positive predictive value of at least one rheumatology-based code diagnostic in children [12, 26]. Together with the high capture between 80–90% for other rheumatic diseases (RA, AS and SLE) in WARDER [8], this suggests robustness of the current data. The Australian health care system relies on a combination of private and public health care providers, but all can refer children to the Children’s hospital in their state. There is no restriction on access to biologicals other than synthetic DMARD resistant disease activity, while for political more than medical reasons, important valuable outpatient data are not collected/registered in Australia and thus not available for study. Our data are drawn from deidentified administrative health data, that represent the clinician’s main discharge diagnoses, but do not have details of clinical, laboratory or imaging findings. The TNFi dispensing data were not individualized, making it impossible to correct for potential confounders and did not allow direct linkage to the deidentified patient data in the WARDER dataset. Thus, we could not determine the direct impact of drug type/usage/switch on admission rates. We used a 10-year look back period to ascertain new cases but cannot exclude not capturing a small group of young children < 6 years diagnosed prior to 1980, possibly contributing to an underestimation during the early 1990s.

## Conclusion

The rates for a first hospital admission for JIA did not change in the period 1990 to 2012, but overall admission rates including for joint injections increased over time. This suggests that there has been limited impact of new management strategies including the significant uptake of TNFi therapy on the need for hospital-based management in JIA in WA.

## Supplementary Information

**Additional file 1:** **Suppl Table 1.** Application form demonstrating requirements to access bDMARD for JIA in Australia. **Suppl Fig 1.** Primary diagnoses in patients (*n*=80) with a first hospital admission where JIA was a co-diagnosis. **Suppl Figure 2**. Arthrocentesis rates over time in hospital admitted patients with Juvenile arthritis in Western Australia in 0 (a) and 2 joint point (b) analyses.

## Data Availability

WA Health is proprietor of this administrative health data dataset. The data that support the findings of this study were used under license from WA Health Data Linkage Branch. Restrictions apply to the availability of these data, but upon reasonable request and following permission of WA Health and WA Data Linkage Branch data are available from the authors.

## Abbreviations

JIA: Juvenile idiopathic arthritis
TNFi: Tumour necrosis factor inhibitor
WA: Western Australia
DDD: Defined daily doses /1000 population/day
CI: Confidence Interval
APC: Annual percentage change
DMARD: Disease-modifying anti-rheumatic drug
WARDER: Western Australia Rheumatic Disease Epidemiological Register
HMDC: Hospital Morbidity Data Collection
ICD-9-CM: International Classification of Diseases version 9, Clinical Modification version
ICD-10-AM: International Classification of Diseases version 10, Australian Modification
PBS: Pharmaceutical Benefits Scheme
ATC: Anatomical Therapeutic Chemical
HREC: Human Research Ethics Committee
