# Supplementary material for: The effect of TNF treatment uptake on incident hospital admission in Western Australia
Source: Pediatr Rheumatol Online J. 2023 Mar 28;21:29. doi: 10.1186/s12969-023-00810-1 (PMC10045824; doi:10.1186/s12969-023-00810-1)

Suppl Table 1 Application form demonstrating requirements to access bDMARD for JIA in Australia


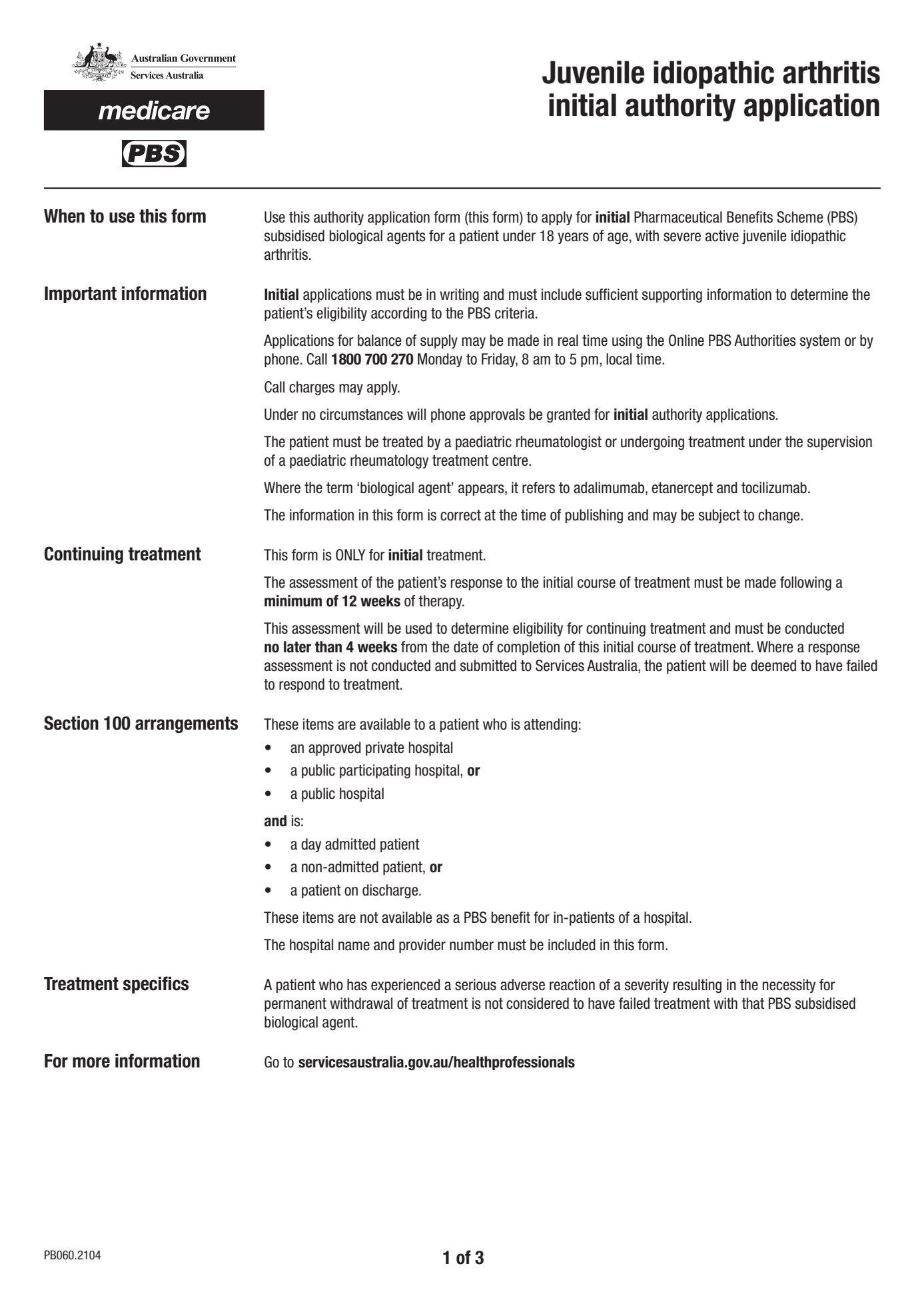


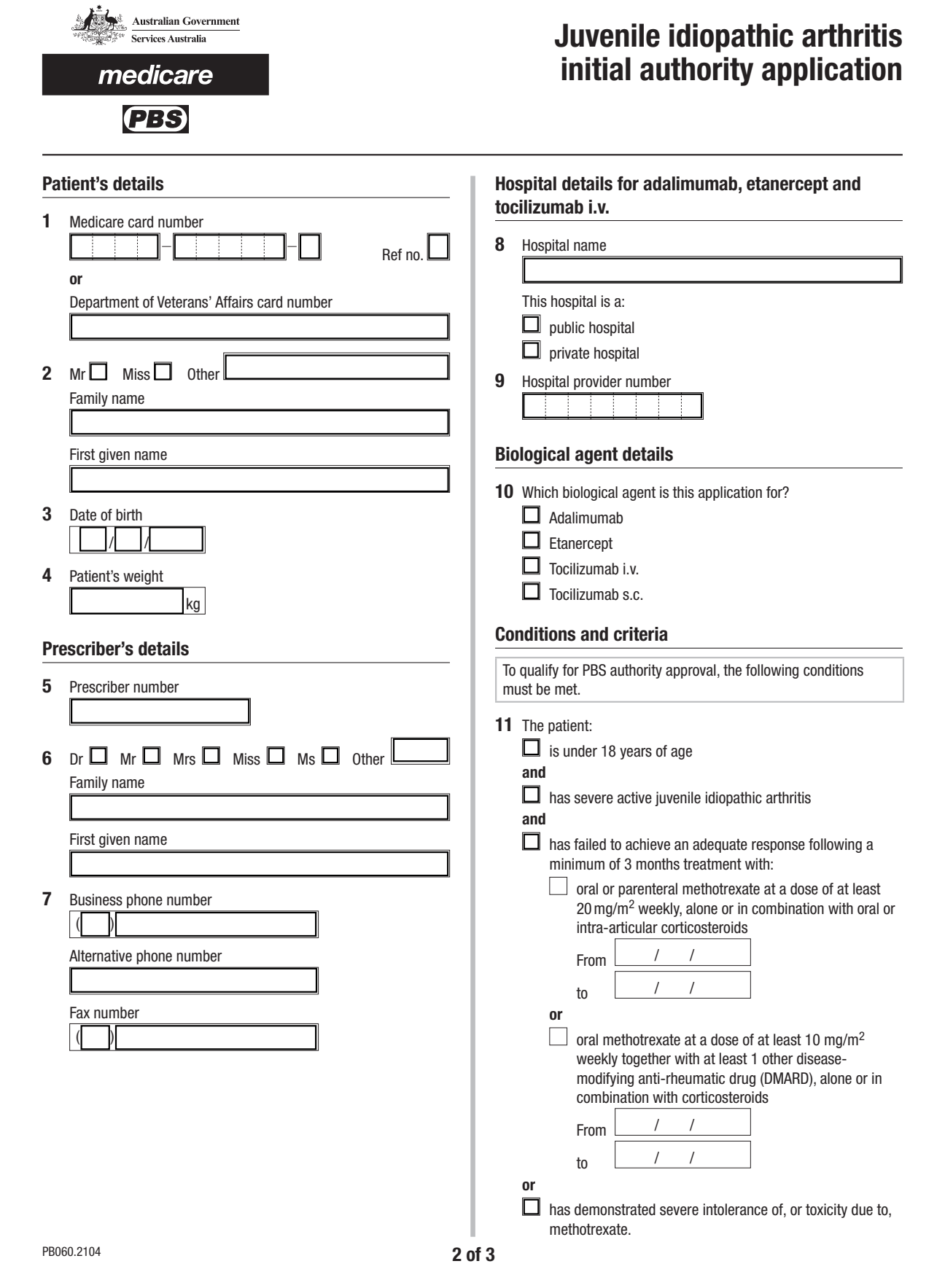

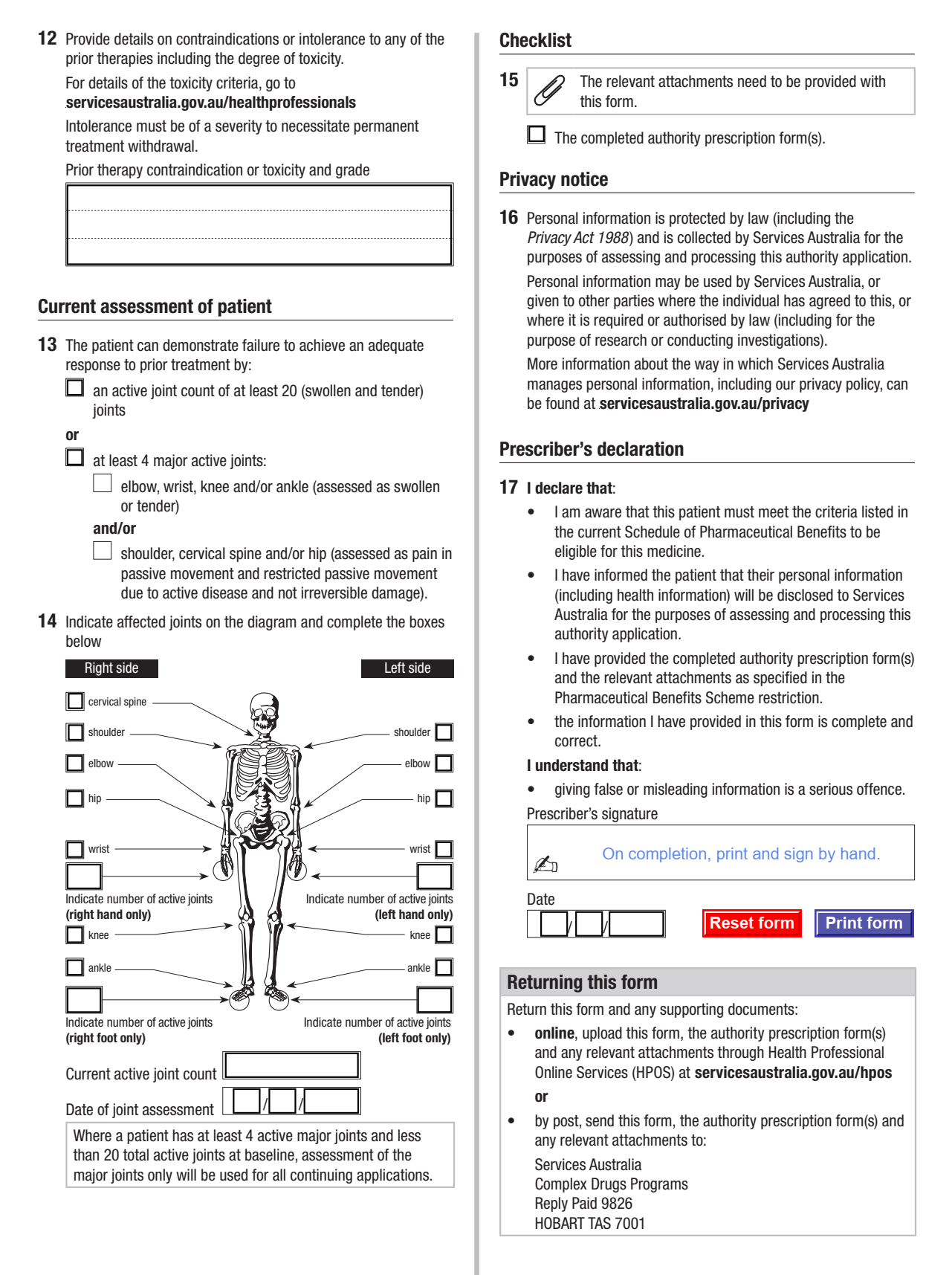


Suppl Fig 1 Primary diagnoses in patients (n=80) with a first hospital admission where JIA was a co-diagnosis.


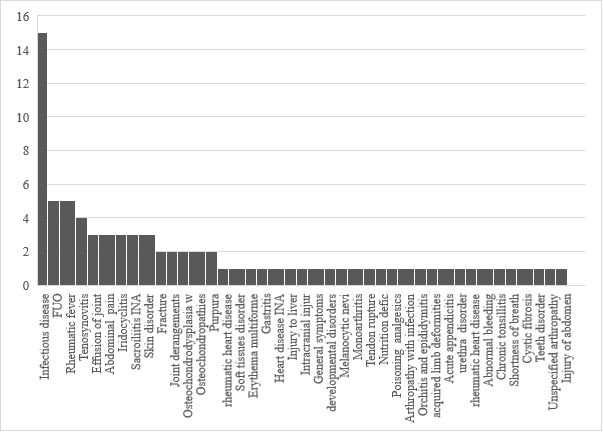


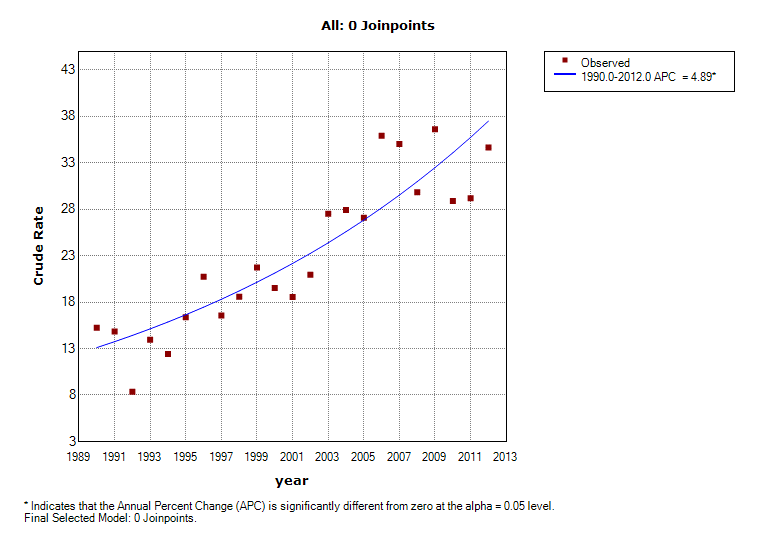
Suppl Figure 2 Arthrocentesis rates over time in hospital admitted patients with Juvenile arthritis in Western Australia in 0 (a) and 2 joint point (b) analyses.


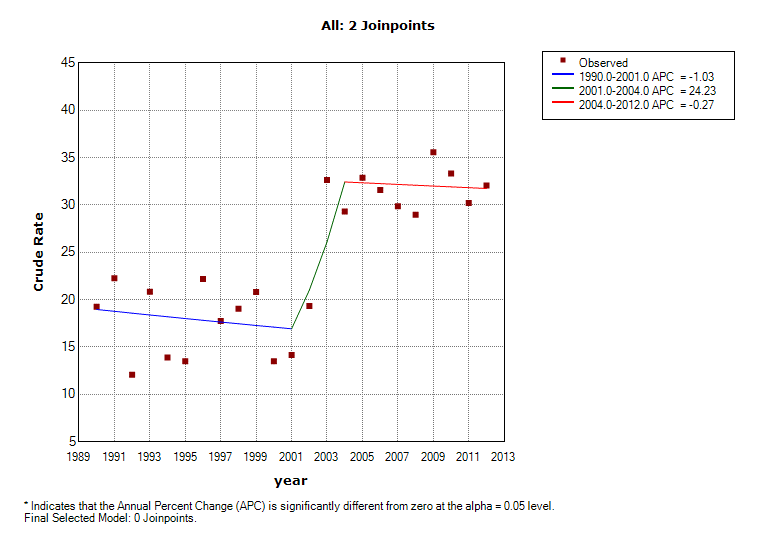

Supplement: Supplementary file 1 — Additional file 1: Suppl Table 1. Application form demonstrating requirements to access bDMARD for JIA in Australia. Suppl Fig 1. Primary diagnoses in patients (n=80) with a first hospital admission where JIA was a co-diagnosis. Suppl Figure 2. Arthrocentesis rates over time in hospital admitted patients with Juvenile arthritis in Western Australia in 0 (a) and 2 joint point (b) analyses. [file 12969_2023_810_MOESM1_ESM.docx]
